# Symmetry-directed complex tessellation of irregular polygons from a single molecular precursor

**DOI:** 10.1039/d6sc00180g

**Published:** 2026-04-29

**Authors:** Wenya Zhai, Zengfu Ou, Ye Chen, Haoyuan Zang, Donghui Guo, Jingcheng Li

**Affiliations:** a Guangdong Provincial Key Laboratory of Magnetoelectric Physics and Devices, School of Physics, Sun Yat-sen University Guangzhou 510275 China guodonghui@mail.sysu.edu.cn; b College of Physics and Electronic Information Engineering, Guilin University of Technology Guilin 541004 China lijch73@mail.sysu.edu.cn

## Abstract

The construction of two-dimensional tessellations using irregular polygonal motifs remains a significant challenge in supramolecular chemistry, primarily due to the difficulty in achieving periodic ordering without intrinsic geometric regularity. Herein, we report the fabrication of molecular tessellations based on two irregular hexagons, highlighting the role of symmetry in the tiling of asymmetric building blocks. By precisely tuning the intramolecular hydrogen-transfer mediated reaction processes, three molecular superlattices are synthesized on a metallic substrate from a single fluorene-based precursor. Scanning tunneling microscopy (STM) measurements demonstrate that all three superlattices are assembled *via* Br-mediated hydrogen bonds, with building blocks adopting irregular hexagonal or decagonal geometries. Structural analysis indicates that one hexagon with mirror symmetry (*C*_s_) and one without intrinsic symmetry achieve tiling by dimerization (*C*_2_ symmetry) or hexamerization (*C*_6_ symmetry) under specific conditions. This work opens a route to constructing complex supramolecular architectures driven by geometric symmetry at the molecular scale.

## Abstract 2

Constructing molecular superlattice tessellations from irregular hexagonal building blocks *via* symmetry-driven dimerization (C_2_) or hexamerization (C_6_) pathways.

## Graphical abstract

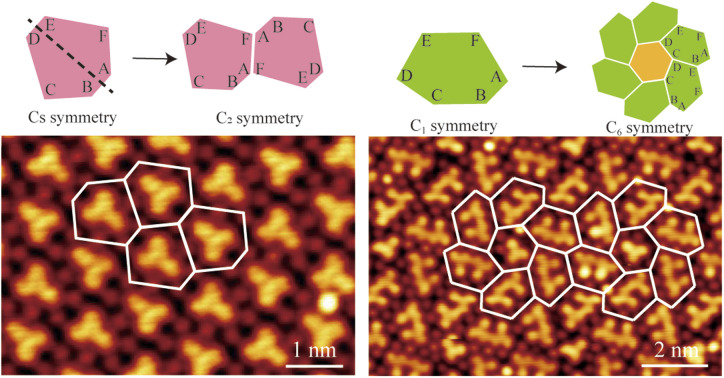

## Introduction

Two-dimensional (2D) molecular tessellation involves the arrangement of molecular building blocks on a plane to form a repeating pattern, like a tiling, without gaps or overlaps. Construction of such supramolecular architectures enables precise tailoring of their physical and chemical properties,^1–4^ a prerequisite for the wide application of molecule-based devices.^5–9^ A primary strategy for fabricating molecular tessellations relies on surface-based chemistry, wherein self-assembly processes drive the formation of nanoscale architectures on substrates.^10^ These self-assembly processes are predominantly governed by intermolecular interactions, such as van der Waals forces, halogen bonding,^11^ hydrogen bonding,^10^ or covalent bonding,^12^ while molecule–substrate interactions may also contribute in specific cases.^13^

Rationally designing molecules with tailored functional groups thus allows control over the architectures of the resulting tessellations. A large variety of molecular tessellations have been successfully constructed on surfaces, encompassing well-defined Archimedean tilings^14–27^ as well as more complex periodic patterns.^28–34^ In these systems, the basic building blocks can be viewed as polygons, with tessellation geometry typically determined by intramolecular bonding. Such bonding defines the vertices of the polygon, and the number of vertices specifies the polygon type. These two factors collectively dictate the overall tiling pattern.^33,35^

To date, most reported molecular tessellations are based on regular polygons, whereas those incorporating irregular polygons remain scarce. For example, three tiling patterns using irregular hexagons have been predicted in theory,^36–39^ with only one type experimentally realized recently.^40^ The reduced symmetry of irregular polygons imposes strict constraints on 2D tessellation formation, rendering their experimental realization highly challenging. In this work, we report a symmetry-directed assembly strategy for constructing tessellation architectures based on two different irregular hexagons. A single molecular precursor, 2,7-dibromo-9-phenyl-9*H*-fluoren-9-ol (DBPFOH) monomer, on Ag(111) was used for the synthesis. By controlling the sequence of debromination and dehydroxylation, molecular superlattices composed of monomers and dimers were formed upon different annealing processes. Notably, the monomers and dimers exhibit irregular hexagonal geometries. Scanning tunneling microscopy (STM) analysis confirms the formation of tessellations with irregular hexagons, illustrating how symmetry and other structural constraints govern the assembly of such complex architectures.

## Results and discussion

**Fig. 1:**
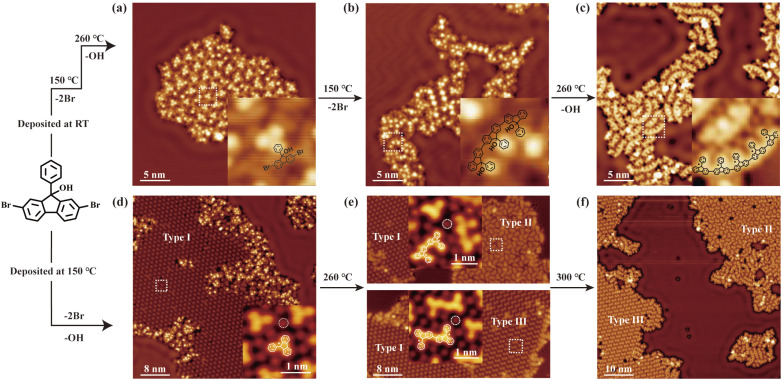
STM images of the formed polymer chains and molecular superstructures from DBPFOH through different annealing processes. (Top panel) STM images of DBPFOH molecules deposited on the Ag(111) substrate kept at room temperature (a) and of polymer chains formed upon annealing the substrate at 150 °C (b) and 260 °C (c), respectively. The inset images in (a–c) show a close-up view of the corresponding highlighted areas (dashed rectangles in white), with chemical models superimposed on top. The black dots on the chemical model in (c) indicate the radicals formed due to the cleavage of OH groups. (Bottom panel) STM images of molecular superstructures formed by DBPFOH molecules after deposition on the Ag(111) substrate held at 150 °C (d), and after annealing at 260 °C (e) and 300 °C (f), respectively. The inset images in (d–f) show the close-up view of the corresponding highlighted areas, with chemical models superimposed on top. The dashed circles indicate the Br atoms. The three distinct phases of the molecular superlattices are labeled as Type I, Type II, and Type III. Arrows in (f) indicate the domains of polymer chains. Tunneling parameters in (a and b): *V* = 100 mV, *I* = 100 pA; (c, d and f): *V* = 100 mV, *I* = 50 pA; (e): *V* = 1 V, *I* = 20 pA.

Fig. 1 illustrates the formation of polymer chains or molecular superlattices from DBPFOH precursors through precisely tuning the reaction pathway on a Ag(111) substrate. Ullmann coupling followed by dehydroxylation of the precursors leads to the formation of polymer chains,^41^ as summarized in Fig. 1(a–c). When evaporated onto the room-temperature substrate, the DBPFOH molecules remain structurally intact, adopting a non-planar configuration.^41^ Their hydroxyl (OH) groups are suspected to be at the molecular top and decoupled from the substrate (Fig. 1(a)). Stepwise annealing of the sample shows that the debromination and dehydroxylation reactions occur at distinct temperatures. Annealing the substrate at lower temperatures first triggers Ullmann coupling, inducing the formation of polymer chains through covalent linking of precursors (Fig. 1(b)). Subsequent annealing promotes dehydroxylation, which cleaves the OH groups from the polymer chains (Fig. 1(c)).

In contrast, DBPFOH molecules evaporated onto the substrate kept at 150 °C resulted in the formation of distinct structures (Fig. 1(d)). A large domain of molecular superlattice is observed in the STM image (referred to as Type I superstructure), wherein polymer chains as shown in Fig. 1(b) decorate its edges. A close-up view of the molecular superlattice shows three-petal-shaped structures, consistent with the backbone structures of DBPFOH molecules (inset in Fig. 1(d)). These three-petal-shaped structures are thus identified as the DBPFOH monomers. Their planar configurations indicate the cleavage of OH groups. The peripheral protrusions (dashed circles in the inset of Fig. 1(d)) are identified as Br atoms. Similar Br-mediated molecular superstructures have been reported previously,^22,27^ including Br-mediated debrominated molecular species forming non-covalent assemblies.^42,43^ Notably, simultaneous debromination and dehydroxylation altered the reaction pathway of DBPFOH precursors compared to the stepwise reaction scenario. The underlying mechanism will be discussed subsequently.

Further annealing of the sample leads to the formation of different molecular superlattices. Upon annealing at 260 °C, the coexistence of three types of molecular superlattices is observed in the STM image (Fig. 1(e)). In addition to the monomer-based Type I molecular superlattice, two new types of molecular superlattices with distinct patterns appear, referred to as Type II (top panel in Fig. 1(e)) and Type III (bottom panel in Fig. 1(e)). The basic building blocks of these new phases are identified as DBPFOH dimers with their OH groups cleaved. There are two types of dimers: one with phenyl groups on the same side (top panel in Fig. 1(e)) and the other with phenyl groups on opposite sides (bottom panel in Fig. 1(e)). Further annealing of the sample at 300 °C removes the Type I molecular superlattice, leaving only Type II and Type III molecular phases. More detailed reaction processes from monomers to dimers with elevated temperatures are summarized in SI Fig. S1–S3. Besides Type II and Type III molecular superlattices, molecular domains with polymer chains coexist on the substrate (Fig. 1(e and f)).

**Fig. 2:**
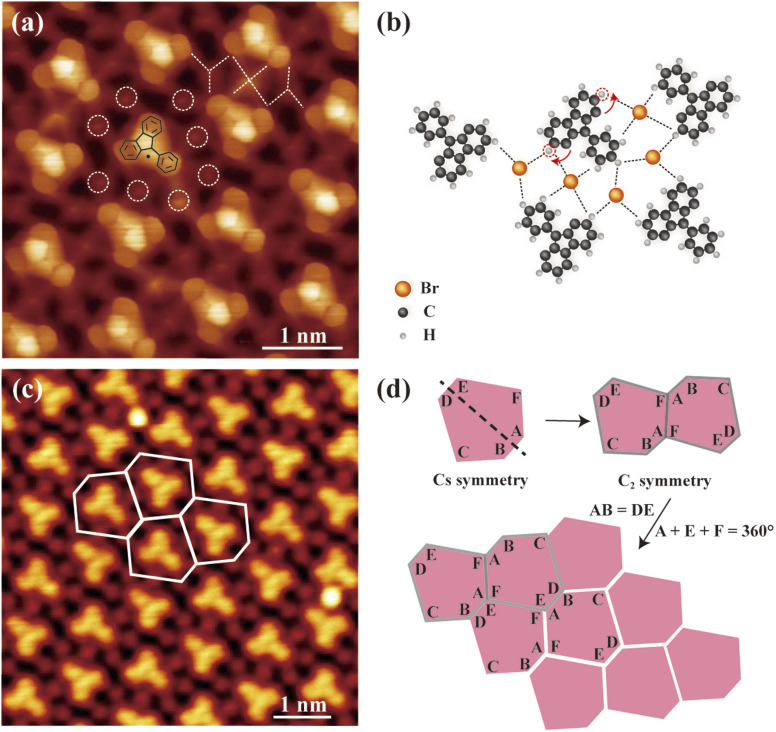
Characterization of 2D tessellation in Type I molecular superlattice. (a) Constant-height STM image (*V* = 2 mV) of a Type I molecular superlattice using a CO-terminated tip. The white dashed lines and circles denote hydrogen bonds and Br atoms, respectively. The chemical model of monomer is superimposed on top. (b) Model structures of a Type I molecular superlattice. Dashed circles highlight the carbon sites of original Br positions in the DBPFOH precursor. The red curved arrows denote intramolecular hydrogen transfer between adjacent carbon sites. Dashed lines indicate the hydrogen bonds. (c) Constant-current STM image (*V* = 100 mV; *I* = 20 pA) of a Type I molecular superlattice, with the irregular hexagonal tiling being highlighted (hexagons in white). (d) Illustration of the underlying mechanism for the irregular hexagonal tiling.

To further characterize the formed molecular superlattices, bond-resolved STM (BR-STM) imaging was performed using CO-terminated tips.^44^Fig. 2(a) displays the BR-STM image of the Type I molecular superlattice. The image clearly resolves the molecular backbone structure, confirming previous identification of the monomer in Fig. 1(d). Each monomer is surrounded by eight Br atoms, as highlighted by white dashed circles in Fig. 2(a) and more clearly seen in Fig. 2(c). Furthermore, the BR-STM image also reveals a rich network of intermolecular interactions (indicated by dashed lines in Fig. 2(a)), indicating hydrogen bonds between Br atoms and molecular monomers.^45,46^ Model structures in Fig. 2(b) detail how neighboring molecules are interlinked *via* Br atoms through hydrogen bonds. Notably, the resolved hydrogen bonds indicate intramolecular hydrogen transfer reactions. The carbon sites highlighted by dashed circles correspond to the original Br positions in the DBPFOH precursor, so no hydrogen should occupy these sites after debromination. The observed hydrogen bonds therefore verify the intermolecular hydrogen transfer from neighboring sites (red curve with arrows), thereby relocating the radical sites from the original Br positions to the adjacent carbon atoms. As a result, Ullmann coupling is suppressed due to induced steric hindrance, and consequently a molecular superlattice is formed instead of polymer chains (SI Fig. S5). The intermolecular hydrogen transfer in single molecules has been well characterized in previous STM measurements^47–49^ and is suggested to mediate on-surface chemical reaction processes^50^ and suppress Ullmann coupling in certain cases.^51^ Our results here provide clear evidence for the intramolecular hydrogen transfer mediated reactions.

The building block of this 2D tessellation can be defined as the monomer with surrounding shared Br atoms, exhibiting the geometry of an irregular hexagon (Fig. 2(c)). Although 2D tessellations of such irregular hexagons have been theoretically predicted, they had not been observed experimentally prior to this study. Structural analysis reveals that the irregular hexagon exhibits mirror symmetry, with sides AB and DE being of equal length (Fig. 2(d)). In contrast to regular hexagons, this irregular hexagon cannot independently tile the entire surface *via* self-translation (SI Fig. S6). However, pairs of these hexagons linked along edges AF(BC) exhibit *C*_2_ rotational symmetry (Fig. 2(d)), facilitating complete surface coverage through tiling. This tiling capability arises from two factors: the mirror symmetry of the hexagon, which leads to the angular sum *A* + *F* + *E* = 360°, and the equality of side lengths AB and DE (Fig. 2(d)). Collectively, these features enable the formation of a continuous, fully covered tessellated surface. The two hexagons connected along the edge CD(EF) also exhibit *C*_2_ symmetry and can tile the surface (SI Fig. S8).

**Fig. 3:**
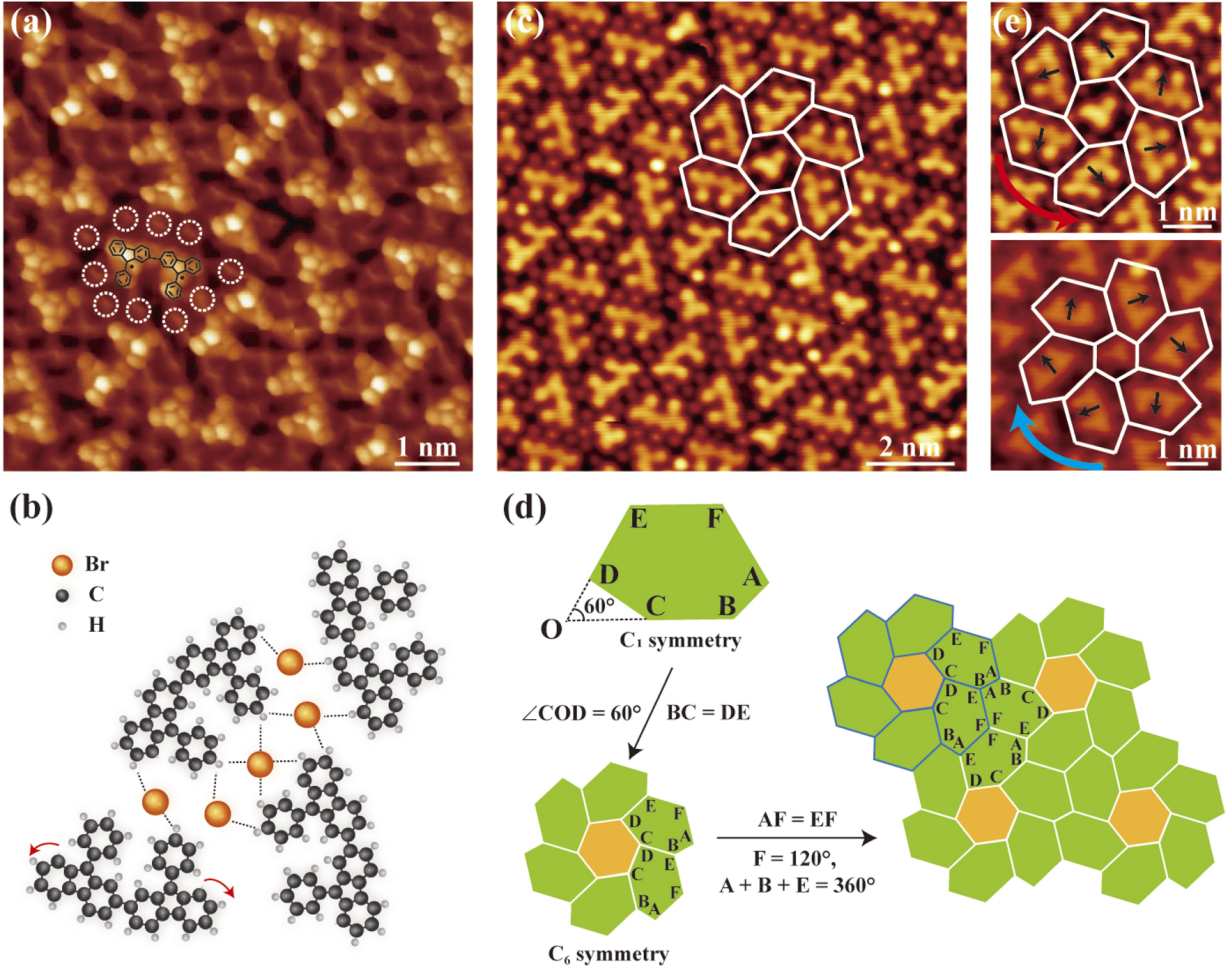
Characterization of 2D tessellation in a Type II molecular superlattice. (a) Constant-height image (*V* = 2 mV) of Type II dimer islands using a CO-terminated tip. The white dashed circles denote Br atoms. The chemical model of dimer is superimposed on top. (b) Model structure of a Type II molecular superlattice. The red curved arrows denote intramolecular hydrogen transfer between adjacent carbon sites. Dashed lines indicate the hydrogen bonds. (c) The STM image (*V* = 600 mV; *I* = 150 pA) of a Type II molecular superlattice, with the tiling pattern highlighted with the white grid (hexagons in white). (d) Schematic diagram of the formation of a flower-like motif and the tiling based on the flower-like motif. (e) Two different chiral conformations for dimers with phenyl groups oriented on the same side. The arrows in black indicate the orientation of phenyl groups. The curved arrows in red (light blue) indicate the chirality (top: *V* = 600 mV and *I* = 150 pA; bottom: *V* = 100 mV and *I* = 50 pA).

For a Type II molecular superstructure, BR-STM imaging reveals the backbone structures of molecular dimers with phenyl groups oriented on the same side (Fig. 3(a)). In a Type I molecular superstructure, we previously demonstrated that intramolecular hydrogen transfer reactions suppress Ullmann coupling. The elevated temperature (260 °C) can overcome the energy barrier for intramolecular hydrogen transfer between the two adjacent carbon sites. Additionally, raising the annealing temperature enhances the surface diffusion of monomers. It is reasonable to deduce that there exists a competitive relationship between Ullmann coupling and intramolecular hydrogen transfer. When Ullmann coupling prevails, molecular dimers are formed, leading to the formation of Type II/III molecular superstructures.

The BR-STM image also reveals complex hydrogen bonding in a Type II molecular superstructure. Each dimer is surrounded by ten Br atoms (Fig. 3(a and b)) and is interlinked *via* hydrogen bonding, as displayed in the model structures in Fig. 3(b). The building block of this 2D tessellation—comprising a dimer surrounded by shared Br atoms—also adopts an irregular hexagonal geometry. However, unlike the Type I molecular superlattice, this irregular hexagon lacks inherent symmetry. Furthermore, this irregular hexagon is also unable to independently tile the entire surface *via* self-translation (SI Fig. S7). Consequently, the tessellation of the Type II molecular superlattice employs a fundamentally distinct tiling mechanism: six dimers and one monomer assemble into a flower-like motif with *C*_6_ symmetry (Fig. 3(c)). The 2D tessellation of a Type II molecular superstructure can be described as the translation of this flower motif.

To elucidate the formation mechanism of the irregular hexagon, we performed a systematic structural analysis. Despite its lack of inherent symmetry, the hexagon exhibits well-defined geometric features that govern its assembly behavior. First, the lengths of sides BC and DE are equivalent, and the angle subtended between these two sides measures 60°. These two geometric constraints collectively facilitate the assembly of six dimers into a flower motif with *C*_6_ symmetry (Fig. 3(d)). Furthermore, three key parameters enable periodic tiling of the flower motif: (i) the sum of angles *A*, *B*, and *E* equals 360°; (ii) angle *F* is fixed at 120°; (iii) sides AF and EF are isometric. This combination of angular and length constraints ensures the formation of extended tiling patterns (Fig. 3(d)). More interestingly, the flower motif exhibits intrinsic chirality. Owing to the unidirectional orientation of phenyl groups on one side of the dimer, chirality emerges upon *C*_6_ assembly of the dimers (Fig. 3(e)). Consequently, the tiling patterns derived from the irregular hexagons in molecular superstructures display two chiral states, extending the current theoretical framework. It is also worth noting that the monomer at the center of the flower motif is not a prerequisite for the formation of the flower motif. At elevated annealing temperatures, molecular superlattices of the same tiling pattern without central monomers are observed (SI Fig. S9), which further corroborates the symmetry-directed formation mechanism proposed by us.

**Fig. 4:**
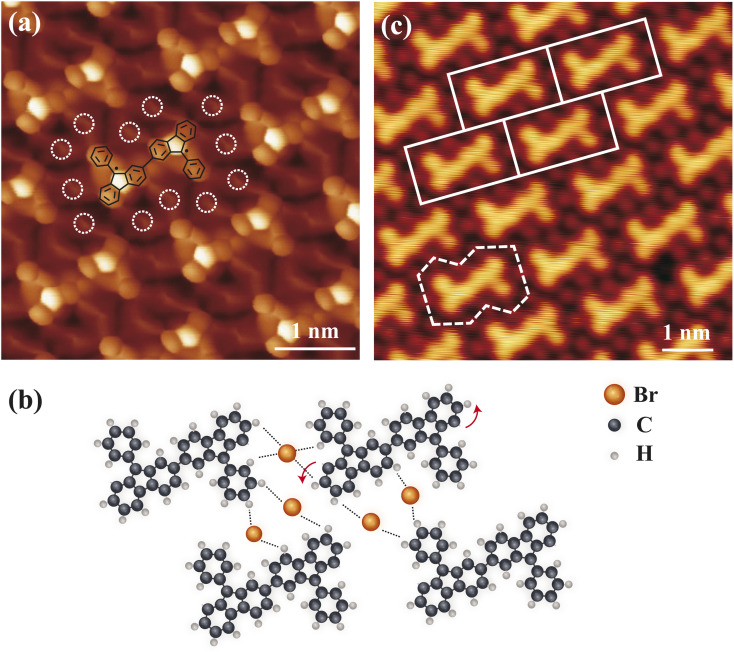
Characterization of 2D tessellation in a Type III molecular superlattice. (a) Constant-height image (*V* = 2 mV) of Type III dimer islands using a CO-terminated tip. The white dashed circles denote Br atoms. The chemical model of dimer is superimposed on top. (b) Model structures of a Type III molecular superlattice. (c) STM image (*V* = 600 mV and *I* = 150 pA) of a Type III molecular superlattice, where the white dashed decagon outlines the fundamental building block and the white solid rectangles indicate the unit cell of the tiling pattern.

When the phenyl groups are positioned on the opposite sides of the dimers, the symmetry of the dimers changes, which results in the formation of the Type III molecular superlattice. The BR-STM image of the molecular superlattice is presented in Fig. 4(a). Similar to the previously discussed Type II molecular superlattices, the dimers are interlinked *via* Br-mediated hydrogen bonds (Fig. 4(b)). The fundamental building block, consisting of the dimeric unit and its surrounding shared Br atoms, can be regarded as an irregular decagonal structural motif (dashed white line in Fig. 4(c)). Since the tiling of the entire structure arises from the periodic translational arrangement of this decagonal motif, the overall molecular superlattice can be categorized as a rectangular lattice (white rectangular in Fig. 4(c)), as the rectangular unit cell already encapsulates all essential symmetry elements of the extended structure. In contrast to previously tiling systems based on irregular hexagonal building blocks, the higher intrinsic symmetry of the rectangular (*D*_2h_) (or decagonal (*C*_2_)) structural motifs simplifies the long-range ordering of the tiling assembly.

## Conclusions

In conclusion, our research demonstrates that intramolecular hydrogen transfer reactions can outcompete Ullmann coupling, thereby regulating the formation of molecular superlattices. Furthermore, we have successfully fabricated molecular tessellations based on irregular polygons, expanding the current library of tessellation architectures beyond conventional regular-polygon-based systems. Most importantly, we introduced a universal symmetry-directed assembly approach that facilitates the construction of ordered 2D tessellations from irregular polygonal motifs. Even if irregular polygons inherently lack symmetry, the innate self-assembly process can still guide them into organized structures by generating symmetry from interconnected units. This study illustrates that symmetry modulation is vital for addressing the geometric complexity of irregular polygon tiling in 2D molecular tessellation.

## Methods

### Experiments

The experimental investigations were conducted using a Unisoku USM1300 ultrahigh-vacuum scanning tunneling microscope system at 5 K. The Ag(111) single crystal (MaTecK GmbH, 99.999%) was cleaned using cycles of Ar^+^ sputtering and subsequent annealing at 550 °C. DBPFOH molecular precursors (Aladdin, 98%) were evaporated from a Knudsen cell kept at 52 °C. Ultrahigh-resolution images were obtained using a CO-functionalized tungsten tip. All STM images were processed using the WSxM software package.^52^

## Author contributions

W. Y. Z. and J. C. L. conceived the project. J. C. L. and D. H. G. supervised the project. W. Y. Z. performed the experiments with the help of Z. F. O., Y. C. and H. Y. Z. W. Y. Z. and J. C. L. analyzed the data and prepared the figures. All authors discussed the results. W. Y. Z. and J. C. L. wrote the manuscript with the help of all authors.

## Conflicts of interest

There are no conflicts to declare.

## Supplementary Material

SC-OLF-D6SC00180G-s001

## Data Availability

The data that support the findings of this study are available from the corresponding author upon reasonable request. Supplementary information (SI): detailed analysis and additional STM images. See DOI: https://doi.org/10.1039/d6sc00180g.
