# Supplementary material for: Symmetry-directed complex tessellation of irregular polygons from a single molecular precursor
Source: Chem Sci. 2026 Apr 29;17(24):11903–10. doi: 10.1039/d6sc00180g (PMC13154872; doi:10.1039/d6sc00180g)
Supplement: SC-017-D6SC00180G-s001 [file SC-017-D6SC00180G-s001.pdf]

Supplementary Information (SI) for Chemical Science.
This journal is © The Royal Society of Chemistry 2026

Supplementary Information for:

Symmetry-Directed Complex Tessellation of Irregular Polygons from a Single Molecular Precursor

Wenya Zhai[a], Zengfu Ou[b], Ye Chen[a], Haoyuan Zang[a], Donghui Guo[a,*], and Jingcheng Li[a,*]

[a] *Guangdong Provincial Key Laboratory of Magnetoelectric Physics and Devices, School of Physics, Sun Yat-sen University, Guangzhou 510275, China*

[b] *College of Physics and Electronic Information Engineering, Guilin University of Technology, Guilin 541004, China*

E-mail: guodonghui@mail.sysu.edu.cn; lijch73@mail.sysu.edu.cn

1. Structural evolution of molecular superlattices by elevated annealing temperatures

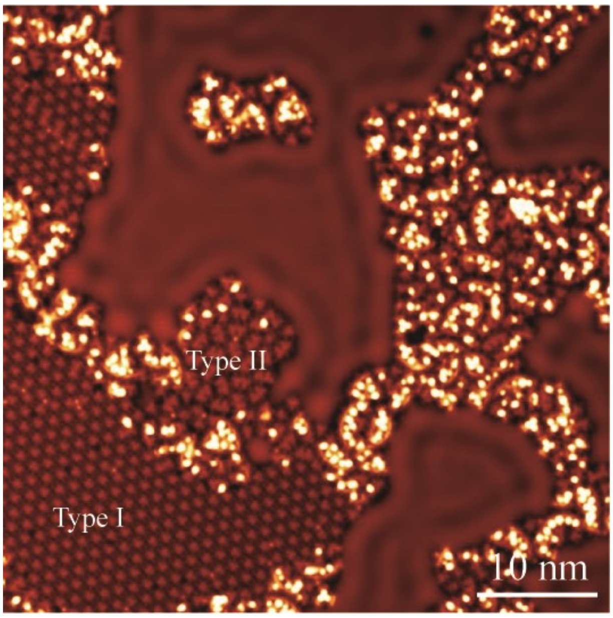

Figure S1: STM images of DBPFOH molecules deposited on Ag(111) substrate kept at 150 °C followed by annealing at 200 °C (V= 100 mV I= 50pA). The surface is dominated by Type I molecular superlattice, with a small amount of Type II coexisting.

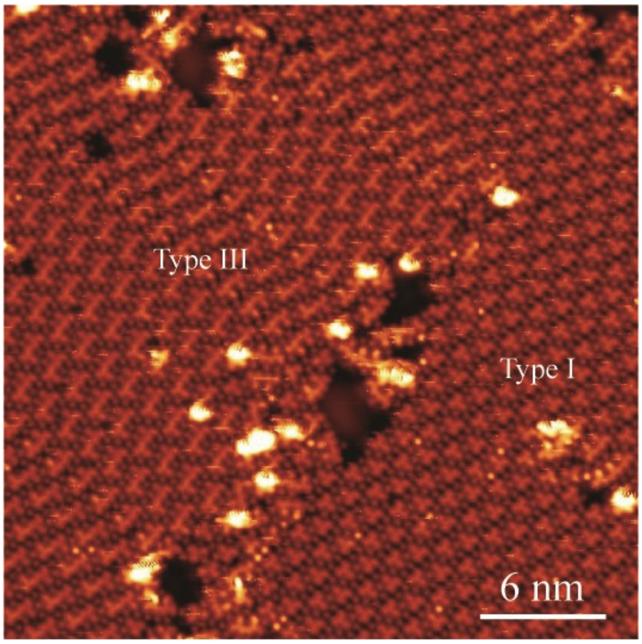

Figure S2: STM images of DBPFOH molecules deposited on Ag(111) substrate kept at 150 °C followed by annealing at 230 °C (V= 100 mV I= 50pA). Type I and Type III (II) molecular superlattices coexist on the surface (only Type III are shown here).

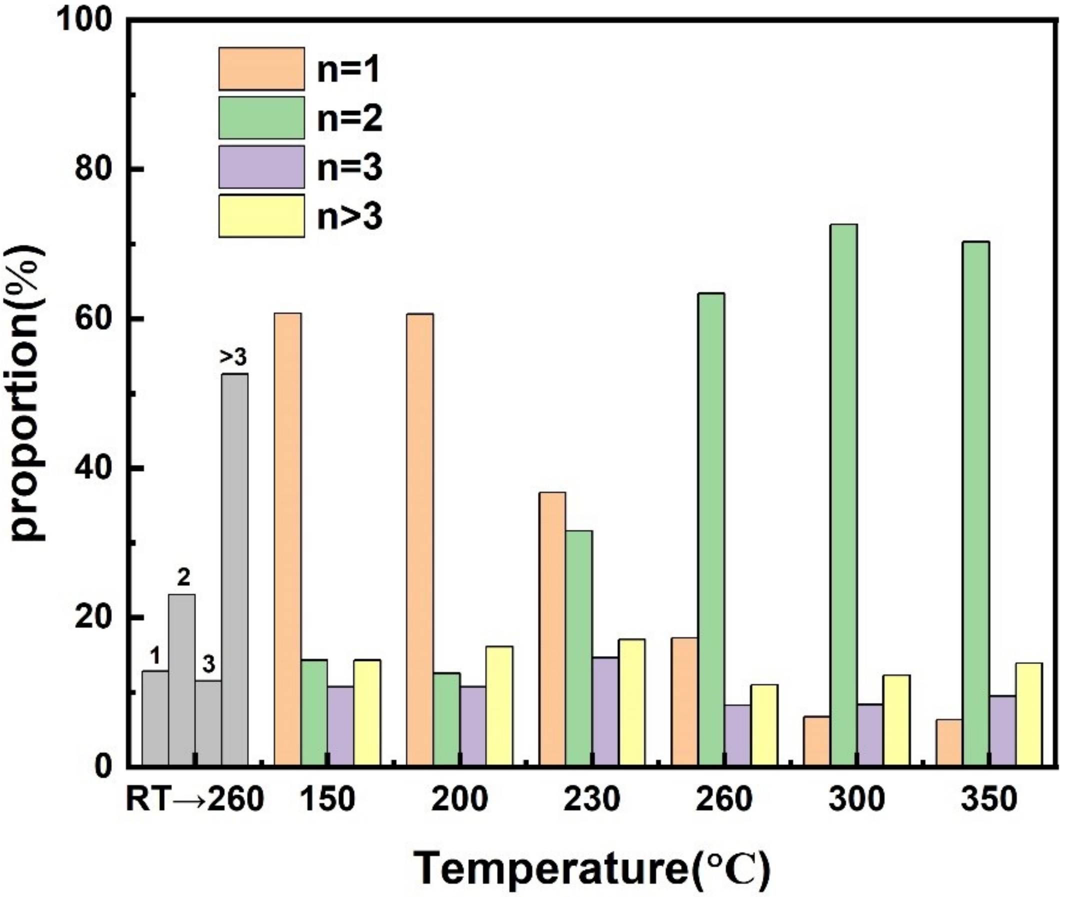

Figure S3: Statistics of monomers, dimers, trimers and long chains under different annealing processes, where n refers to the number of precursor molecules, n = 1: monomers; n = 2: dimers; n = 3: trimers; n > 3: long chains. Gray bars are results from the samples that DBPFOH molecules are deposited on Ag(111) substrate kept at room temperature followed by annealing at 260 ℃. Colored bars are results from the samples that DBPFOH molecules are deposited on Ag(111) substrate kept at 150 ℃ followed by stepwise annealing as noted in the figure. (The statistical data for each temperature are derived from six 200 × 200 nm^2 STM images, covering a total sampled area of 0.24 um^2.)

2. BR-STM image of Type I, Type II, Type III superlattice with model structures imposed

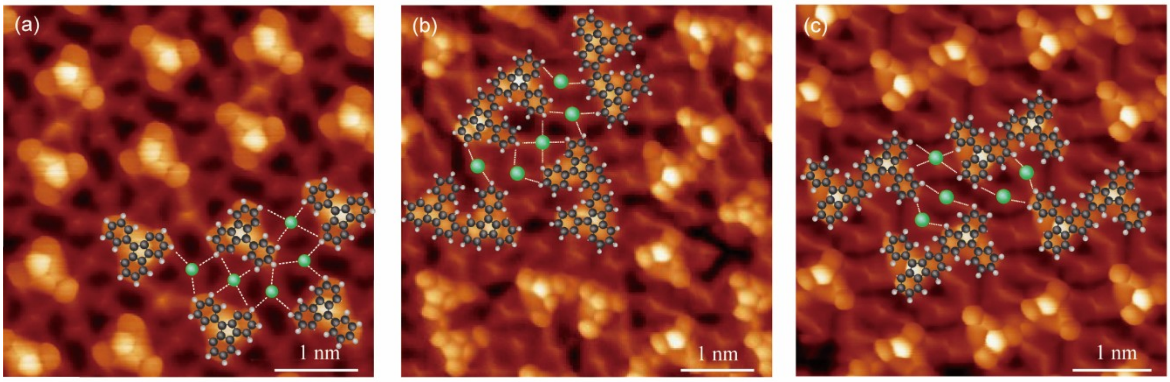

Figure S4: Zoom-in BR-STM images of Type I (a), Type II (b) and Type III (c) molecular superlattices with model structures imposed on top (V=2 mV).

3. Intramolecular hydrogen transfers mediated reaction pathway

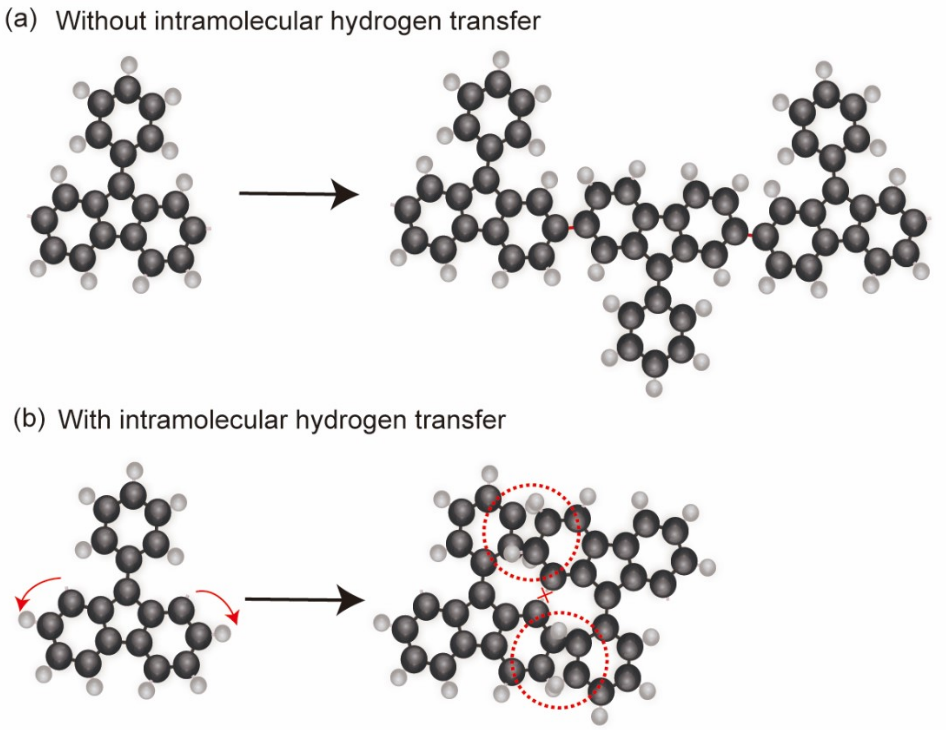

Figure S5: Reaction pathways of DBPFOH on Ag(111): (a) Ullmann coupling occurs when no intramolecular hydrogen transfer is involved (indicated by red bonds); (b) intramolecular hydrogen transfer suppresses Ullmann coupling due to steric hindrance (indicated by dashed circles).

4. Single irregular hexagons fail to tile

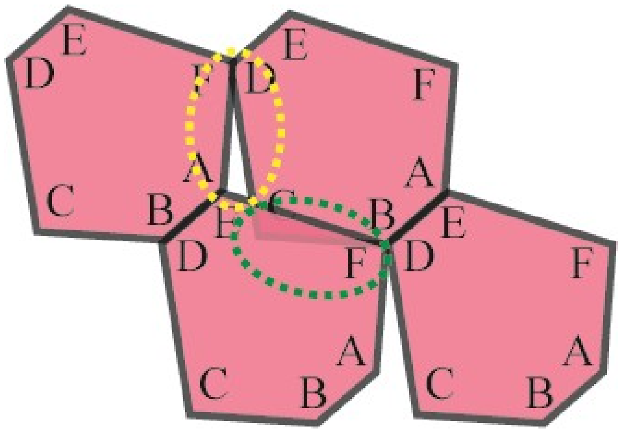

Figure S6: Irregular hexagons with Cs symmetry (Type I in the manuscript) fail to form a continuous tiling via self-translation, resulting in gaps (indicated by dashed yellow circles) and overlaps (indicated by dashed green circles) in their packing arrangement.

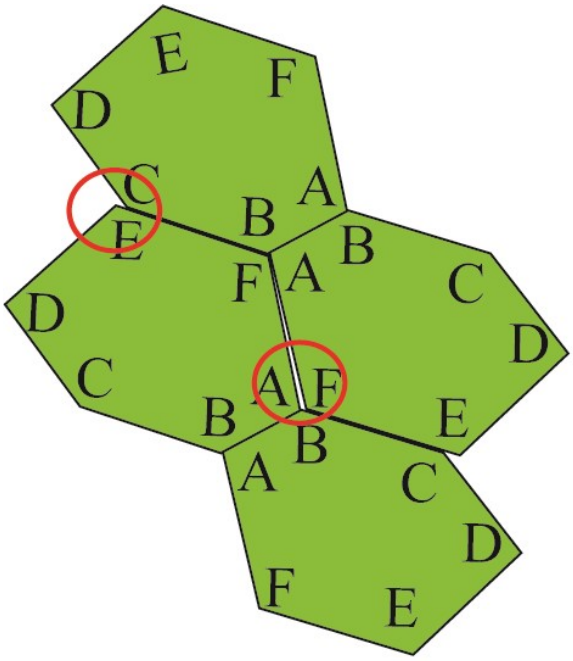

Figure S7: Irregular hexagons with no symmetry (Type II in the manuscript) fail to form a continuous tiling via self-translation, resulting in gaps (indicated by red circles) in their packing arrangement.

5. Two hexagons (Type I) connected along the edge CD(EF)

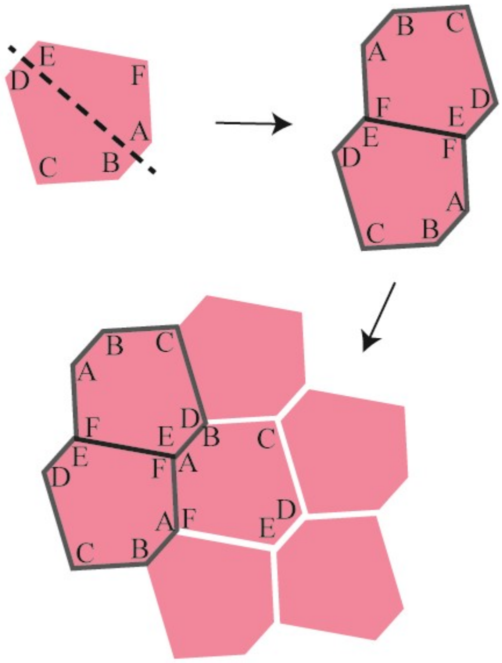

Figure S8: Two hexagons (Type I) connected along the edge CD(EF) exhibit C_2 symmetry and can tile the surface.

6. Tiling of flower motif without central monomer in type II configuration

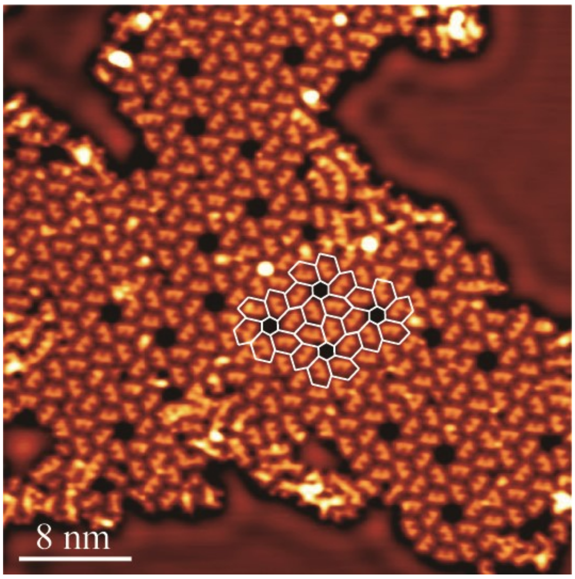

Figure S9: STM image of Type II configuration without a central monomer (V= 100mV, I= 50pA). Monomers disappear upon further annealing of the sample (Figure 3 in manuscript) to 300 °C, yet the characteristic tiling pattern remains.
